# Granulocyte colony-stimulating factor-induced hypersensitivity reaction with leukocytosis in a pediatric germ cell tumor patient: a case report

**DOI:** 10.3389/fped.2026.1749442

**Published:** 2026-06-08

**Authors:** Yuhui Tan, Chaoyong Wei, Wenyan Xiao, Yilan Fu, Xi Chen, Yanxiang Tang, Changli Tang

**Affiliations:** 1 Department of Pharmacy, Xichang People's Hospital, Xichang, Sichuan, China; 2 Department of Thoracic and Cardiovascular Surgery, Xichang People's Hospital, Xichang, Sichuan, China; 3 Department of Pharmacy, Hunan Cancer Hospital, Changsha, Hunan, China; 4 Department of Gynecological Oncology, Hunan Cancer Hospital, Changsha, Hunan, China

**Keywords:** child, efbemalenograstim alfa-vuxw, febrile neutropenia, filgrastim biosimilar, granulocyte colony-stimulating factor, hypersensitivity, leukocytosis

## Abstract

**Background:**

Chemotherapy-induced bone marrow suppression significantly increases the risk of febrile neutropenia (FN) in cancer patients. Granulocyte colony-stimulating factor (G-CSF) is a cornerstone therapy for FN prophylaxis and treatment that promotes myeloid progenitor cell proliferation and differentiation, thereby reducing the duration of neutropenia. While G-CSF is generally well tolerated and has a favorable safety profile, rare but life-threatening adverse events may occur.

**Case presentation:**

We present the case of a 14-year-old female with a germ cell tumor who developed a systemic hypersensitivity reaction following prophylactic administration of efbemalenograstim alfa-vuxw (20 mg/dose) after her third chemotherapy cycle. Notably, the patient had previously tolerated two full doses of efbemalenograstim alfa-vuxw and one full dose of short-acting G-CSF (filgrastim biosimilar 5 μg/kg) without any adverse events during the first two chemotherapy cycles. Within two hours post-injection, she exhibited severe hypotension (74/52 mmHg), hypoxemia (SpO₂ 81%), and marked leukocytosis (55.32 → 104.25 × 10⁹/L within 24 h). Emergency intervention with epinephrine and dexamethasone resolved the symptoms. Peripheral blood analysis revealed eosinophilia (0.16 × 10⁹/L), suggesting drug sensitization. During the fourth chemotherapy cycle, subcutaneous recombinant human G-CSF (rhG-CSF, filgrastim biosimilar; 5 μg/kg) was administered, but the patient experienced a nearly identical reaction within 30 min (BP 53/29 mmHg, SpO₂ 97%). The temporal correlation and clinical consistency confirmed a G-CSF-induced systemic hypersensitivity reaction.

**Conclusion:**

To our knowledge, this represents the first documented pediatric case of recurrent systemic hypersensitivity reactions induced by sequential administration of different G-CSF formulations following chemotherapy. Notably, long-acting G-CSF (efbemalenograstim alfa-vuxw) was associated with both hypersensitivity and pronounced leukocytosis. Our findings highlight the following: 1. Hypersensitivity to G-CSF, although rare, requires heightened clinical vigilance; 2. The Substitution of G-CSF products may not preclude recurrent reactions; 3. The safety profile and optimal dosing of efbemalenograstim alfa-vuxw in pediatric populations warrant further validation in controlled trials.

## Introduction

Malignant ovarian germ cell tumors (MOGCTs) are rare cancers that affect mainly teenage girls and young women (1). Recent data reveal an incidence of approximately 6 cases per million among 14-year-olds and 27 cases per million among females aged 15−19. Notably, malignant ovarian germ cell tumors account for a higher proportion of all ovarian malignancies in Asian and African populations (approximately 15%) compared to Western countries (approximately 5%) (2). While chemotherapy remains the standard treatment, it often causes severe neutropenia and fever that require close monitoring.

Granulocyte colony-stimulating factor (G-CSF), with three main formulations available, has become essential for preventing febrile neutropenia. The first-generation filgrastim, a nonglycosylated recombinant human G-CSF, was the initial exogenous G-CSF approved for clinical use (3), with over 30 years of clinical experience (4). Biosimilars made with filgrastim have reduced costs but require daily injections, impacting patient compliance. Second-generation pegfilgrastim improved convenience through PEG modification for longer action. The newest third-generation efbemalenograstim alfa-vuxw, developed in China, uses Fc fusion technology for extended duration. Globally, G-CSF has demonstrated favorable tolerability in both adult and pediatric populations (5–7), with a low incidence of hypersensitivity reactions. Bone pain remains the most frequently reported adverse effect.

We report the case of a 14-year-old patient with a germ cell tumor who developed systemic hypersensitivity reactions (HSRs) to two different G-CSF formulations after two chemotherapy cycles. The long-acting efbemalenograstim alfa-vuxw induced both HSR and leukocytosis. Although G-CSF-related HSR (8) and leukocytosis (9, 10) have been reported separately, this is the first pediatric case demonstrating concurrent HSR and leukocytosis following sequential use of distinct G-CSF preparations postchemotherapy. Given the rarity of this clinical phenomenon, we analyzed the potential mechanisms of G-CSF-induced HSR and clinical management strategies on the basis of current evidence, along with possible causes of leukocytosis induced by efbemalenograstim alfa-vuxw in this pediatric case.

## Case presentation

This case involves a 14-year-old female with a documented allergy to cefazolin and cefuroxime but no other significant medical history who was diagnosed with stage IIIA1 left ovarian dysgerminoma. Following surgical treatment, she underwent four cycles of BEP chemotherapy, receiving 15 U/m^2^ bleomycin on day 1, 167 mg/m^2^ etoposide on days 1–3, and 33 mg/m^2^ cisplatin on days 1–3 per cycle. Prior to the first chemotherapy cycle, the patient received a temporary subcutaneous injection of filgrastim biosimilar (brand name: Terjin®, manufacturer: Xiamen Amoytop Biotech Co., Ltd.) at 5 μg/kg due to leukopenia (2.55 × 10⁹/L) and neutropenia (1.8 × 10⁹/L), with no adverse reactions observed. For the subsequent two chemotherapy cycles, prophylactic subcutaneous injections of efbemalenograstim alfa-vuxw 20 mg per dose (brand name: Ryzneuta®, manufacturer: Evive Biotech Co. Ltd.) were routinely administered 48 h post-chemotherapy to prevent febrile neutropenia. During this period, only reactive leukocytosis (peak 38.59 × 10⁹/L) was noted, without other clinical abnormalities.

At the start of her third chemotherapy cycle, the patient's baseline vital signs included a temperature of 36.8 °C, a heart rate of 133 bpm, a respiratory rate of 20/min, and blood pressure of 101/80 mmHg. Prechemotherapy labs revealed leukocytosis (21.77 × 10⁹/L) attributed to prior G-CSF stimulation after excluding infection. At 24 h post-chemotherapy, her WBC count was 9.56 × 10⁹/L, and her neutrophil count was 8.72 × 10⁹/L, demonstrating a rapid decline from baseline. Given this trajectory, prophylactic efbemalenograstim alfa-vuxw was administered at the 48-hour mark. Within two hours of electrolyte infusion, she developed acute agitation, nausea, vomiting, and syncope, with clinical findings of tachycardia (150 bpm), tachypnea (24/min), hypotension (91/52 mmHg), and hypoxemia (SpO_2_ 81%) consistent with anaphylaxis. Initial management with intramuscular metoclopramide (10 mg) and intravenous dexamethasone (10 mg) did not include epinephrine, deviating from standard anaphylaxis guidelines. This approach failed to prevent clinical deterioration, with hypotension worsening to 75/49 mmHg. The patient required transfer to the intensive care unit, where she ultimately stabilized after receiving norepinephrine (8 mg by continuous intravenous infusion), intramuscular epinephrine (0.5 mg), and along with high-flow oxygen. Blood samples drawn shortly after dexamethasone administration revealed acute leukocytosis (WBC 55.32→104.25 × 10⁹/L), granulocytosis (102.71 × 10⁹/L), and basophilia (0.16→0.41 × 10⁹/L). Subsequent serial monitoring revealed a spontaneous downward trend in both white blood cell and neutrophil counts, and no further clinical intervention was initiated.

**Figure 1:**
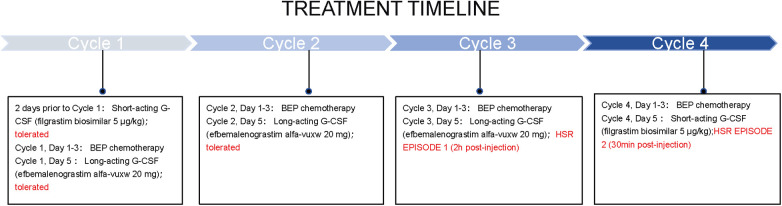
Antitumor treatment regimen and granulocyte colony-stimulating factor (G-CSF) administration schedule. The timeline illustrates the chronological sequence of the patient's chemotherapy cycles, explicitly indicating the timing, specific drug name, and administered dosage for each G-CSF injection.

**Figure 2:**
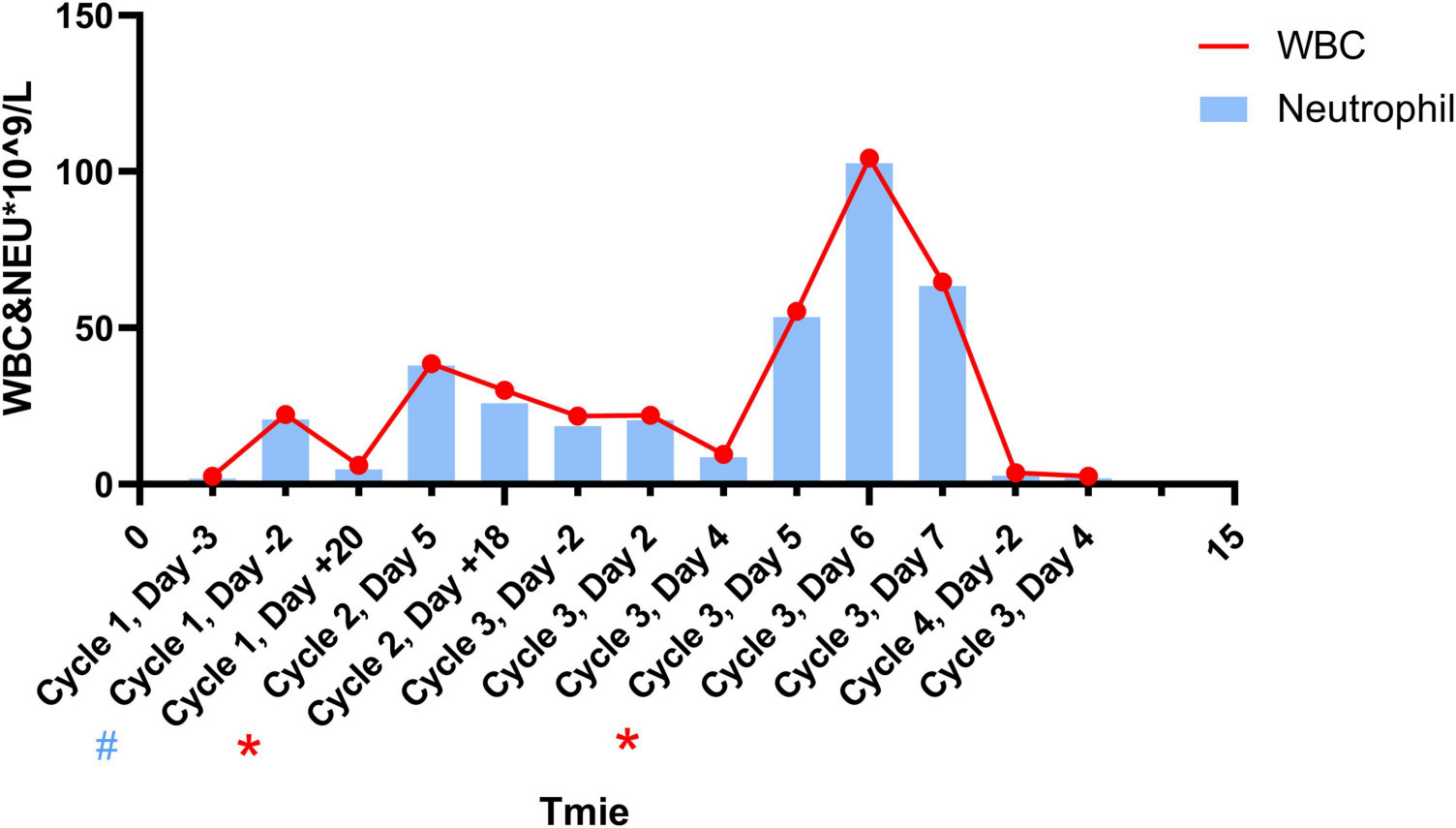
Serial measurements of white blood cell (WBC) and neutrophil counts (NEU) across all treatment cycles. Blue symbols indicate time points of short-acting filgrastim biosimilar administration with corresponding laboratory data; red symbols indicate time points of efbemalenograstim alfa-vuxw administration with corresponding laboratory data. *X*-axis labels follow the convention “Cycle X, Day Y,” where Day 1 is defined as the first day of chemotherapy for each cycle; negative values indicate days prior to chemotherapy initiation. Two administration events (efbemalenograstim alfa-vuxw at Cycle 1, Day 5, and filgrastim biosimilar at Cycle 4, Day 5) are not plotted due to the absence of immediate post-administration laboratory data.

At the start of her fourth chemotherapy cycle, the patient's vital signs at admission were recorded as follows: temperature, 36.7 °C; pulse, 131 bpm; respiratory rate, 18/min; and blood pressure, 106/73 mmHg. Three days post-chemotherapy, laboratory evaluation revealed leukopenia (WBC 2.45 × 10⁹/L) accompanied by neutropenia (NEU 1.94 × 10⁹/L), which prompted the administration of a filgrastim biosimilar (Terjin®, Xiamen Amoytop Biotech) at a dose of 5 μg/kg. Approximately 30 min after administration, the patient experienced recurrent symptoms, including chest tightness, palpitations, nausea, and transient visual disturbances. Physical examination revealed severe tachycardia (175 bpm), tachypnea (21/min), and critical hypotension (53/29 mmHg), although oxygen saturation remained stable at 98%. Immediate intervention with intramuscular epinephrine (0.5 mg) and antihistamines led to successful resolution of symptoms, with no residual effects observed. Due to the acute nature of this hypersensitivity event and the focus on emergent hemodynamic stabilization, a post-administration complete blood count was not obtained during this episode. Table 1 compares the two G-CSF formulations and summarizes the reaction profiles. The antitumor treatment regimen and G-CSF administration schedule are presented in Figure 1. Figure 2 shows G-CSF use and associated leukocyte and neutrophil dynamics across all treatment cycles.

**Table 1:** Characteristics of G-CSF preparations and associated hypersensitivity reactions.

G-CSF drug	Active ingredient	Excipients	Onset after administration	Clinical manifestations
efbemalenograstim alfa-vuxw	Recombinant Human G-CSF Dimer; Fc Fragment of Human IgG2 Immunoglobulin	Glacial acetic acid, Sodium acetate, Sorbitol, Polysorbate 20, Edetate disodium (Disodium EDTA)	2 h	Restlessness, nausea, perioral cyanosis, vomiting, transient syncope; HR 150 bpm, RR 24/min, SpO₂ 100%, BP 74/52 mmHg
rhG-CSF（filgrastim biosimilar）	Human G-CSF (*Escherichia coli*-derived)	Mannitol, Sodium acetate, Acetic acid, Polysorbate 80	30 min	Palpitations, chest tightness, nausea/vomiting, transient blurred vision; Max HR 175 bpm, RR 21/min, SpO₂ 97%, BP 53/29 mmHg

Fortunately, all planned chemotherapy cycles were completed without further complications. Post-chemotherapy leukopenia was subsequently managed with supportive granulocyte-stimulating agents, including leucogen (sodium leucogenin, a synthetic small-molecule agent), Diyu Shengbai tablets (Sanguisorba officinalis extract), and Compound Zaofan pills (a traditional Chinese medicine preparation containing melanterite, Panax quinquefolius, Hippocampus spp., Cinnamomum cassia, and jujube).

## Discussion

While recombinant G-CSF has intrinsic allergenic potential as a foreign protein (11), reported hypersensitivity reactions occur significantly less frequently than with other biologics, especially monoclonal antibodies (12). According to a systematic review by Bumbăcea et al., only 40 cases of G-CSF-associated hypersensitivity have been documented (13), ranging from mild cutaneous reactions to severe cardiovascular collapse, including 9 cases fulfilling WAO grade 5 anaphylaxis criteria (13, 14). Our patient presented with the classic the WAO grade 5 manifestations (hypotension accompanied by transient loss of consciousness), which was further supported by a Naranjo score of 8, suggesting probable causation (15).

This case has exhibited two clinically significant features. First, the patient demonstrated similar hypersensitivity to both the *E. coli*-derived filgrastim biosimilar (16) and the Chinese hamster ovary (CHO) cell line-produced efbemalenograstim alfa-vuxw, despite their distinct manufacturing platforms and excipient compositions (polysorbate 80 vs. 20) (17). Notably, the fourth-cycle reaction occurred after isolated administration of filgrastim without any concomitant medications. Second, we observed an unprecedented association with extreme leukocytosis (peak WBC count 104.25 × 10⁹/L). Together, these findings suggest that the native G-CSF structure itself, rather than production-related variables, excipient components, or co-administered compounds, is the primary antigenic trigger. Notably, neither Bumbăcea's comprehensive analysis (13) nor our thorough literature review identified similar cases of G-CSF-induced systemic hypersensitivity coinciding with such pronounced leukocytosis.

Beyond these immunological observations, this case also highlights critical gaps in anaphylaxis preparedness. Despite clear signs of severe hypersensitivity, including hypotension, hypoxemia, tachycardia, and syncope, intramuscular epinephrine, the guideline-recommended first-line treatment, was not administered during the initial response. Instead, metoclopramide and dexamethasone were used, which are not appropriate primary therapies for acute anaphylaxis. The rapid progression to refractory hypotension requiring intensive care may have been mitigated by earlier epinephrine administration.

Equally important, this case underscores the need for robust patient and caregiver education on anaphylaxis recognition and emergency response. Patients receiving G-CSF, particularly those with a prior hypersensitivity history, and their families should receive structured training on the early signs of anaphylaxis. In well-resourced settings, prescribing an epinephrine auto-injector for home use is the standard of care and should be strongly considered when G-CSF is self-administered outside the hospital. However, the authors acknowledge that epinephrine auto-injectors may not be universally available, particularly in resource-limited or primary care settings. Under such circumstances, alternative strategies represent a pragmatic and potentially life-saving safeguard—for example, ensuring that the first dose of any new G-CSF formulation is administered in a monitored clinical environment with conventional epinephrine ampoules and trained personnel immediately available. These measures, whether high-tech or low-tech, aim to reduce the risk of severe outcomes should a recurrent hypersensitivity reaction occur.

Apart from highlighting the inadequacies in preparedness for allergic reactions, this case also prompted further reflection on the potential mechanisms underlying G-CSF–-induced allergic reactions. Emerging evidence suggests that biologics-induced hypersensitivity reactions may involve multiple pathophysiological mechanisms beyond classical IgE-mediated type I reactions, including cytokine release syndrome and mixed reaction patterns (18). G-CSF-associated hypersensitivity appears to exhibit similar mechanistic diversity. For example, PEG-conjugated pegfilgrastim has been reported to induce hypersensitivity through both IgE-dependent and complement-mediated pathways (19), whereas *E. coli*-derived products might trigger mast cell activation through residual host protein contaminants (20). Although third-generation efbemalenograstim alfa-vuxw utilizes CHO cell expression and glycosylation modifications that could theoretically reduce immunogenicity (21), our case indicates that sensitization risk may persist in certain individuals, possibly owing to genetic predisposition.

These observations have several clinical implications. First, patients with previous G-CSF hypersensitivity reactions may require close monitoring even when switching to formulations from different production platforms. Second, as efbemalenograstim alfa-vuxw is relatively new to the market, comprehensive pharmacovigilance and large-scale studies will be essential to characterize its rare adverse effect profile fully. Current clinical guidelines do not advocate routine skin testing for G-CSF hypersensitivity, given its low incidence. However, for high-risk patients with a prior history of hypersensitivity reactions to biologic agents, skin testing may still serve as a reasonable precautionary measure. It should be noted that the clinical reliability of subcutaneous pretesting remains limited, as even patients with confirmed filgrastim hypersensitivity may yield negative results (13, 18). Therefore, establishing standardized rapid drug desensitization (RDD) protocols for this patient population warrants urgent clinical attention, given the essential therapeutic role of G-CSF in oncology care.

Beyond the allergic reaction and associated prevention strategies, the extreme elevation in white blood cell count observed in this case merits thorough exploration. In the present case, efbemalenograstim alfa-vuxw administration was associated with both systemic hypersensitivity and marked leukocytosis (WBC ≥ 100 × 10⁹/L). While similar leukocytosis has been reported in pediatric patients receiving PEG-rhG-CSF (9), this appears to be the first documented occurrence with efbemalenograstim alfa-vuxw. This unique pharmacodynamic response may be attributed to its innovative molecular design: (1) a dimeric G-CSF structure conjugated to human IgG2 Fc via a peptide linker (22), which reduces renal clearance while preserving bioactivity; (2) the absence of PEGylation-related activity attenuation (23), maintaining full G-CSFR binding affinity; and (3) Fc‒FcRn interactions enabling pH-dependent lysosomal evasion and extended plasma half-life (24–26).

Beyond the drug's intrinsic pharmacodynamic properties, the rapid onset of extreme leukocytosis—occurring within hours of administration—is mechanistically more consistent with neutrophil demargination from the vascular endothelial pool than with accelerated granulopoiesis, which typically requires several days to manifest (27). This interpretation is further supported by the concurrent administration of dexamethasone during the acute hypersensitivity episode, as corticosteroids independently induce neutrophil demargination by downregulating endothelial adhesion molecules (28). The observed leukocytosis may therefore represent a combined demargination effect of G-CSF and corticosteroid co-administration, rather than being attributable solely to the hypersensitivity reaction or to increased bone marrow production. Clinically, recognizing this distinction can help avoid unnecessary investigations for infection or hematological malignancy when unexpected leukocytosis occurs in this context.

Efbemalenograstim alfa-vuxw has received regulatory approval in multiple jurisdictions, including China and the United States, for the prevention of chemotherapy-induced febrile neutropenia in adults with nonmyeloid malignancies (29, 30). However, its use in pediatric populations presents several clinical challenges. While the adult dosage is well established at a fixed 20 mg subcutaneous dose per cycle, pediatric dosing remains undefined because of insufficient clinical trial data. This contrasts with PEG-rhG-CSF, which has established pediatric applications (6, 31, 32), albeit with significant international variation in dosing strategies, including FDA-recommended weight-based dosing, Japanese guidelines specifying a fixed 3.6 mg dose (equivalent to adult recommendations) (33), and Chinese protocols recommending 100 mcg/kg (maximum 6 mg per dose). These regional differences reflect the principles of precision medicine in pediatrics, accounting for ethnic variations in body composition and locally derived pharmacodynamic data. Pediatric hematopoietic physiology presents unique considerations, including greater red marrow volume (compared with that of adults, where yellow marrow comprises ∼70% by age 25) (34), enhanced bone marrow microenvironment activity, and increased hematopoietic sensitivity. These physiological factors suggest that the standard 20 mg adult dose of efbemalenograstim alfa-vuxw may produce exaggerated hematopoietic stimulation in children, potentially amplified by the drug's Fc-fusion structure. A cautious, stepwise dose titration approach with intensive hematologic monitoring may therefore be preferable to fixed dosing in pediatric patients. The drug's pharmacokinetic profile—particularly its prolonged half-life and high receptor affinity—while beneficial in adults, may require special consideration in children. There is an urgent need for dedicated pharmacokinetic studies, evidence-based pediatric dosing guidelines, and safety and efficacy evaluations in younger populations. These knowledge gaps highlight the critical importance of targeted clinical research to establish optimal risk‒benefit parameters for efbemalenograstim alfa-vuxw use in pediatric oncology.

This case has several notable limitations. First, the lack of serum tryptase testing and cutaneous allergy testing precluded definitive classification of the hypersensitivity reaction as IgE-mediated or non-IgE-mediated, a limitation that reflects the current clinical practice of insufficient routine monitoring for low-immunogenicity G-CSF agents (13). Second, as discussed above, the extreme leukocytosis observed in this case likely reflects the combined demargination effects of G-CSF and concurrently administered dexamethasone. While this interpretation is mechanistically plausible, the retrospective nature of this case precludes definitive separation of each contributing factor. Prospective studies with serial pre- and post-medication blood sampling would be valuable to quantify the relative contribution of each agent to the observed leukocytosis. Third, the current fixed 20 mg adult dose of efbemalenograstim alfa-vuxw may produce exaggerated hematopoietic stimulation in pediatric patients, highlighting the need for dedicated pediatric dosing studies.

## Conclusion and outlook

In summary, we report the first documented pediatric case of a systemic hypersensitivity reaction following the administration of two distinct G-CSF formulations, with marked leukocytosis specifically observed after efbemalenograstim alfa-vuxw administration. This case has three important clinical implications. First, although rare, G-CSF-induced systemic hypersensitivity may develop rapidly with potentially life-threatening severity. Second, switching between formulations may not prevent recurrent hypersensitivity reactions, suggesting possible cross-reactivity between structurally different G-CSF products. Third, the markedly elevated white blood cell count observed in this case may be attributed to the combined demargination effects of G-CSF and corticosteroids, rather than solely to increased granulocyte production. However, the individual contribution of each factor cannot be clearly distinguished at present and warrants further clarification through prospective studies. These observations highlight the importance of administering G-CSF in controlled medical settings with appropriate postdose monitoring (≥ 60 min) and immediate access to emergency interventions. Moreover, they emphasize the critical need for dedicated pediatric clinical trials to establish the safety profile and optimal dosing strategy for efbemalenograstim alfa-vuxw in children.

## Data Availability

The original contributions presented in the study are included in the article/Supplementary Material, further inquiries can be directed to the corresponding authors.
